# VEXAS Syndrome Presenting With Pleuritis

**DOI:** 10.1002/ccr3.71590

**Published:** 2025-12-02

**Authors:** Makoto Ito, Yasushi Murakami, Michita Suzuki, Takahiko Yasuda, Norio Takagi

**Affiliations:** ^1^ Department of Hematology Tokoname City Hospital Tokoname Japan; ^2^ Department of Hematology and Oncology Nagoya University Graduate School of Medicine Nagoya Japan; ^3^ Department of Respiratory Medicine Tokoname City Hospital Tokoname Japan; ^4^ Department of Rheumatology National Hospital Organization Nagoya Medical Center Nagoya Japan; ^5^ Clinical Research Center National Hospital Organization Nagoya Medical Center Nagoya Japan

**Keywords:** myelodysplastic syndrome, pleuritis, UBA1 mutation, VEXAS syndrome

## Abstract

Elderly men with pleuritis, systemic inflammation, cytopenias, and bone‐marrow vacuolization may have VEXAS due to somatic *UBA1* mutations. Pulmonary involvement, including pleuritis, can be a key presentation despite the absence of classic chondritis, and early recognition may enable timely initiation of corticosteroid therapy.

## Graphical abstract

Chest CT showing pleural effusion and pleural thickening in a patient with recurrent pleuritis (A), and bone marrow smear demonstrating vacuolated myeloid precursors characteristic of VEXAS syndrome (B).

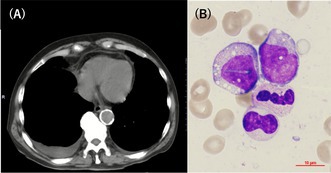

## 1 Case Presentation

**FIGURE 1:**
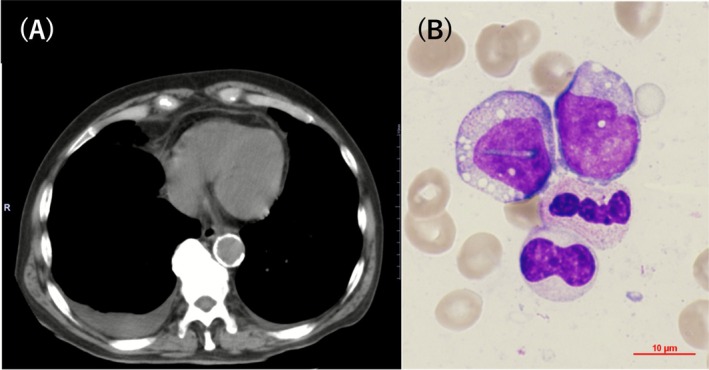
Images at the time of admission. Chest CT on admission revealed right‐predominant pleural effusion (A). Bone marrow examination showed cytoplasmic vacuoles in myeloid precursors (B).

**FIGURE 2:**
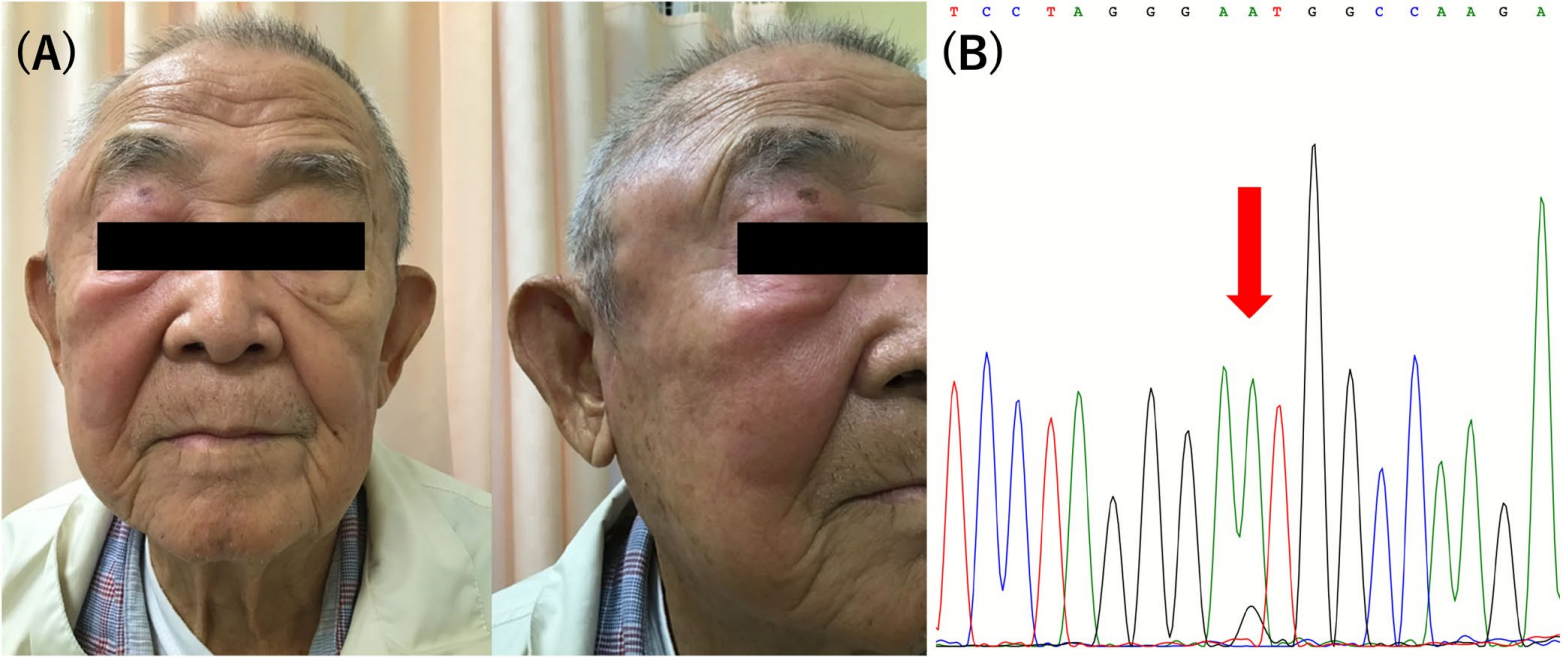
Clinical pictures and genetic analysis one month after presentation. Erythema extending from the right temporal region to the face, photographed from frontal and right oblique angles (A). Sanger sequencing of peripheral blood revealed a p.Met41Val (c.121A > G) mutation, indicated by the red arrow (B).

An 80‐year‐old man was referred for persistent low‐grade fever and right‐sided chest pain lasting 1 month. He had been taking fenofibrate (320 mg/day) for hyperlipidemia and amlodipine (5 mg/day) for hypertension for over 20 years. He had a 20‐cigarette‐per‐day smoking history from age 20 to 60. Diagnostic evaluation revealed bilateral pleural effusion, predominantly on the right side (Figure 1A), elevated C‐reactive protein level (11.73 mg/dL), and pancytopenia (white blood cell count 3300/μL, hemoglobin 7.7 g/dL, mean corpuscular volume 102.6 fL, platelet count 129,000/μL). Bone marrow examination revealed myelodysplastic syndrome (MDS) with low blasts and cytoplasmic vacuoles in myeloid precursors (Figure 1B). One month later, the patient developed erythema extending from the right temporal region to the face (Figure 2A) and tracheal chondritis, evidenced by hoarseness, anterior neck tenderness, CT demonstrating swelling of the epiglottic region, and direct visualization with fiberoptic laryngoscopy by an otolaryngologist. VEXAS was suspected because of the triad of refractory systemic inflammation, hematologic abnormalities with MDS, and characteristic myeloid vacuolization, together with new‐onset chondritis in an elderly man, thereby prompting targeted UBA1 testing. A somatic *UBA1* p.Met41Val mutation was identified in peripheral blood using Sanger sequencing (Figure 2B), confirming vacuoles, E1 enzyme, X‐linked, autoinflammatory, somatic (VEXAS) syndrome [1]. Prednisolone therapy, initiated at 0.5 mg/kg/day, markedly improved pleuritis and normalized the white blood cell count and hemoglobin level despite persistent mild thrombocytopenia (white blood cell count 5000/μL; hemoglobin 14.4 g/dL, platelet count 87,000/μL). Pleuritis improved both clinically (resolution of pleuritic chest pain) and radiologically, with follow‐up chest CT demonstrating complete resolution of the pleural effusion. Prednisolone was gradually tapered and continued at 3 mg/day, with sustained remission for 2 years.

## 2 Discussion

VEXAS syndrome is a recently identified adult‐onset disease caused by somatic mutations in *UBA1*. Although cartilage inflammation is a hallmark of VEXAS syndrome, pulmonary involvement is increasingly recognized [2]. Pulmonary involvement in VEXAS includes parenchymal disease, mediastinal adenomegaly, and pleural effusions [2]. The frequency of pulmonary lesions varies across studies, but pleuritis is especially uncommon, reported in about 2% of VEXAS patients with pleuropulmonary manifestations [2]. This case initially presented with pleuritis and subsequently developed tracheal chondritis. Alternative diagnoses—including infection (tuberculous and bacterial), malignancy‐ or heart‐failure–related effusions, pulmonary embolism, and autoimmune/vasculitic disease—were considered and not supported by clinical, imaging, microbiological, and serological evaluations; the subsequent chondritis and sustained steroid response were consistent with VEXAS. Thoracentesis was not performed due to the small volume, and we acknowledge this as a limitation. Clinicians should carefully evaluate disease features, including inflammatory status and coexisting MDS, and monitor symptoms associated with VEXAS syndrome.

Treatment of VEXAS syndrome remains challenging, with no consistently effective therapies established to date. The p.Met41Val variant is typically associated with a poorer prognosis, and steroid therapy is generally ineffective in resolving VEXAS‐related anemia. Nevertheless, this patient notably responded to prednisolone, achieving significant anemia resolution. Clinicians should consider VEXAS syndrome in elderly male patients presenting with systemic inflammation, pleuritis, and anemia; early recognition may enable timely initiation of corticosteroid therapy.

## Author Contributions

**Makoto Ito:** conceptualization, data curation, formal analysis, investigation, methodology, project administration, resources, visualization, writing – original draft. **Yasushi Murakami:** conceptualization, data curation, resources, supervision, visualization, writing – review and editing. **Michita Suzuki:** conceptualization, resources, supervision, writing – review and editing. **Takahiko Yasuda:** data curation, formal analysis, methodology, resources, software, visualization, writing – review and editing. **Norio Takagi:** conceptualization, project administration, supervision, writing – review and editing.

## Ethics Statement

All specimens were acquired with written informed consent from the patient, in compliance with the Declaration of Helsinki. Consent for publication in print was also obtained from the patient.

## Data Availability

The data that support the findings of this study are available within the article. Additional de‐identified clinical information is not publicly available due to privacy restrictions, but may be shared by the corresponding author upon reasonable request and subject to institutional and patient privacy policies.
